# Identifying Patterns of Discontinuing and Recommencing Pre-exposure Prophylaxis in the Context of Sexual Behavior Among Gay and Bisexual Men in Australia

**DOI:** 10.1007/s10461-023-04013-3

**Published:** 2023-02-22

**Authors:** Steven P. Philpot, Dean Murphy, Curtis Chan, Bridget Haire, Nathanael Wells, Doug Fraser, Andrew E. Grulich, Benjamin R. Bavinton

**Affiliations:** 1 Level 6, Wallace Wurth Building, High St, Kensington, NSW 2052 Australia; 2 The Kirby Institute, UNSW Sydney, Sydney, Australia; 3 Department of Infectious Diseases, Alfred Hospital and Central Clinical School, Monash University, Melbourne, Australia

**Keywords:** Gay and bisexual men, PrEP, Qualitative, Discontinuation, Recommencement, Patterns

## Abstract

We mapped gay and bisexual men’s (GBM) patterns of using pre-exposure prophylaxis (PrEP) over time and explored sexual behavior as PrEP use changed. We conducted semi-structured interviews between June 2020 and February 2021 with 40 GBM living in Australia who had changed their PrEP use since initiating. There was considerable diversity in patterns of discontinuation, suspension, and recommencement of PrEP. Reasons for changing PrEP use mostly centered on accurate perceived changes to HIV risk. Twelve participants reported condomless anal intercourse with casual or fuckbuddy partners after discontinuing PrEP. These sex events were unanticipated, condoms were not a preferred option, and other risk reduction strategies were applied inconsistently. Service delivery and health promotion can support safer sex among GBM when PrEP use fluctuates by promoting event-driven PrEP and/or non-condom-based risk reduction methods during periods off daily PrEP, and guiding GBM to better recognize changing circumstances of risk and when to recommence PrEP.

## Introduction

Pre-exposure prophylaxis (PrEP) is highly effective at preventing HIV acquisition, including among gay, bisexual, and other men who have sex with men (GBM) [1]. As PrEP has moved from trials to implementation, attention has turned to how GBM incorporate PrEP into their lives. Many GBM do not view PrEP as a permanent feature of their lives and cycle on and off it according to ‘seasons of risk’, alongside perceptions of their likelihood to engage in HIV risk behavior [2–5]. However, much research has concentrated solely on PrEP discontinuation [2, 6]. HIV acquisition has occurred in some GBM who perceived they were at less risk of HIV and discontinued PrEP [7–9]; thus, discontinuation has been identified as a problem for HIV transmission. Studies have found variable but substantial discontinuation of PrEP among GBM, usually framed around non-retention in care or (lack of) overall PrEP coverage in priority populations [2, 10–19]. However, although acknowledging that discontinuation of PrEP is only concerning if HIV risk is ongoing, most studies typically do not take into account GBM’s sexual behavior after discontinuation and instead focus on rates or causes of discontinuation.

A limitation associated with examining PrEP discontinuation disconnected from GBM’s sexual behavior is that such assessments provide little context as to whether GBM remain at risk of acquiring HIV. To circumvent this limitation, Harberer et al. (2015) developed the concept of ‘prevention-effective adherence’ [20], whereby the focus is on whether an individual correctly adheres to PrEP around the times of potential sexual exposure to HIV *as well as* whether they adopt other HIV risk reduction tools if they do not use PrEP (such as condoms, serosorting [21], or accurate knowledge of a partner’s undetectable viral load). This approach takes into consideration whether an individual’s discontinuation of PrEP is concurrent with engagement in HIV risk behavior. A recent study found that among Australian GBM, prevention-effective adherence was very high at 99% [22].

Researchers have acknowledged that GBM are likely to change their PrEP use over time owing to personal, social, structural, and/or economic factors. However, few studies have explored GBM’s patterns of PrEP use beyond merely reporting on rates of one occasion of discontinuation, masking nuances of GBM’s ongoing use of PrEP. Exceptions have found that that some GBM PrEP users did not practice linear and continued use, but that they anticipated they would continuously discontinue and recommence PrEP according to their level of risk [3], that about one-fifth of the sample recommended PrEP after each occasion they discontinued [9], and that a large minority of participants had discontinued PrEP at least twice, a few up to five occasions [18]. Gaining insight into patterns of PrEP use will contribute to understanding the decisions GBM make around when to use PrEP or not, and the contexts of HIV risk and sexual behavior as PrEP use changes. In Australia, PrEP and associated medical care have been publicly subsidized through Australia’s universal healthcare system, Medicare, since April 2018. Awareness of PrEP among GBM suitable for PrEP is high at 87% [24] and nearly fifty thousand men had accessed PrEP as of September 2021 [25]. The Australian context provides an opportunity to explore how GBM use PrEP over time in a setting of high accessibility and uptake. We describe GBM’s patterns and practices of PrEP use, including contexts of discontinuation and recommencement, as well as exploring HIV risk behavior alongside changing PrEP use.

## Methods

Semi-structured interviews were conducted with 40 GBM living in Australia between June 2020 and February 2021. Ethical approval was received from the University of New South Wales Human Research Ethics Committee (HC200377) and ACON (202018).

### Recruitment

Eligibility criteria included: Identification as a man; identification as gay or bisexual or participation in sex with at least one man in the previous year; being at least 18 years of age; having a self-reported HIV-negative status; living in Australia; and being able to participate in English. Participants also needed to have changed their PrEP use in some way since first initiating it, including discontinuing and/or switching dosing regimens.

Participants were recruited in two ways. First, men who were in a prior PrEP clinical trial [7] and had given consent to be contacted for future research opportunities were emailed and invited to click a link to express their interest in participating. Second, this link was promoted by a HIV and LGBTIQ community organization in emails and Facebook posts. Eligible participants were then contacted by a member of the research team to arrange an interview. We did not have capacity to offer participants compensation for their participation and participants were told this on the participant information sheet before they signed consent.

### Data Collection

Semi-structured interviews lasted between 45 and 90 min, and were audio-recorded and then transcribed verbatim and de-identified. Participants were informed when arranging interviews (and on their participant information sheet) that due to COVID-19 restrictions, their interview would be conducted online via a video platform. When interview times were being arranged, participants were told they could have their video turned off during their interview. All participants elected to have their video on. All interviews were conducted by SP. Interviews explored how and why participants initiated, discontinued, and recommenced PrEP, and if they had switched dosing regimens, how and why they did so. The interviews also explored how men’s sexual behaviors changed alongside changing PrEP use.

### Analysis

Interviews were analyzed using a codebook style of inductive thematic analysis [26]. The process began with a close reading of each transcript to ensure familiarity with the data. Transcripts were then imported into NVivo version 12 for coding. As each transcript was re-read, recurring patterns from the data were categorized into an initial coding framework. The codebook style usually calls for early development of themes, but our strategy involved an initial development of themes and then perpetual revisions to themes as the coding framework developed. For this paper, data regarding PrEP discontinuation and recommencement, and concurrent sexual behavior are reported using a largely descriptive approach to organization of themes. All analyses were conducted by SP, a qualitative researcher with extensive experience researching the needs of gay and bisexual men. SP identifies as a white gay man who has used PrEP. This positioning provided insider knowledge of PrEP use but was carefully and constantly considered so as not to misunderstand or make assumptions about participants’ perspectives. The broader research team included researchers experienced in the sexual health and PrEP needs of GBM, who provided feedback throughout the analysis process where needed. While SP conducted analyses, the research team met on multiple occasions to discuss and interpret the data and the coding framework as it developed.

In this paper we report on ‘discontinuing’ PrEP as distinguishable from ‘suspending’ PrEP because they were identified as meaningfully distinct during analysis. We defined ‘discontinuing’ as stopping PrEP for the foreseeable future with no *intention* of recommencing any time soon (though many did ultimately recommence). Conversely, ‘voluntary suspending’ was defined as temporary time off PrEP during periods of anticipated absent sex (with an intention to recommence), mostly in the context of COVID-19 restrictions in 2020 and 2021 limiting sexual opportunities, or when travelling for work or to visit family and friends. ‘Involuntary suspending’ was defined as temporary time off PrEP that was unexpected and unavoidable, due to depleted PrEP supply while travelling or negotiating a significant mental health issue. These categories were identified inductively during the analysis process.

## Results

### Sample

The demographics of the 40 participants are presented in Table 1. Ages ranged from 23 to 71 years with a median age of 39. Most (n = 37) identified as gay. Most (n = 32) reported their cultural/ethnic background as White European, one South Asian, two Southeast Asian, and five Northeast Asian. Only two participants did not have access to Medicare as they were not permanent residents in Australia at the time of their interview. The sample was highly educated, with 24 having university education. Most participants (n = 28) accessed their PrEP prescription from a doctor, nine from a sexual health clinic, and three were using pills from a clinical trial. The majority (n = 34) accessed pills from a local pharmacy.

**Table 1:** Demographic characteristics

Demographic	Number (*n* = 40)
Age	
18–25	2
26–30	9
31–35	10
36–45	3
46–55	7
56–65	8
> 66	1
Ethnic/cultural background	
White European	32
South Asian	1
Southeast Asian	2
Northeast Asian	5
Sexual identity	
Gay	37
Bisexual	2
Queer	1
Education	
Secondary	10
Trade certificate/Diploma	6
Undergraduate degree	13
Postgraduate degree	11
Employment	
Full time	33
Part time	1
Student	6
Retired	3

**Fig. 1:**
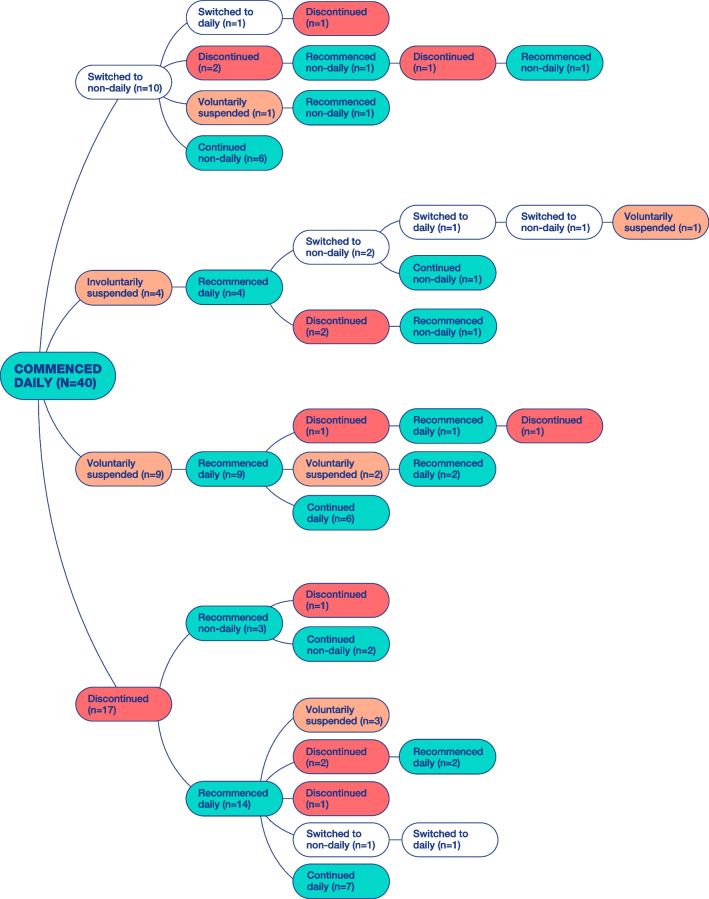
Patterns of PrEP use over time

All participants initiated PrEP with daily dosing, 37 as part of a clinical trial of daily PrEP that ran between March 2016 and April 2018. Prior to using PrEP, half (n = 20) said they frequently used condoms, nine said they frequently used condoms with casual partners but not with regular partners (i.e. a boyfriend or fuckbuddy), and 11 said they used condoms infrequently. The majority reported that after initiating PrEP, their condom use dramatically decreased with all sexual partners. At *any* point during their PrEP use, 22 had discontinued, 13 had voluntarily suspended, and 4 had involuntarily suspended at least once (Fig. 1). This paper describes participants who had discontinued/suspended and recommenced PrEP. For further detail of participants who had switched dosing regimens, please refer to Philpot et al. 2022 [27]. As visualized in Fig. 1, discontinuing PrEP was rarely a permanent or fixed decision, even if at the time of discontinuing participants had no intention to recommence. Most who discontinued had recommenced at least once; several more than once. Importantly, Fig. 1 identifies the true complexity and diversity of how participants used PrEP over time, indicating the intricacy of some GBM’s ongoing PrEP pathways.

In the following analysis, we highlight three descriptive themes: discontinuing PrEP; condomless anal intercourse (CLAI) after discontinuing or suspending PrEP; and PrEP recommencement. Each of these themes complements and adds detail to our presentation of participants’ overall patterns of PrEP use as visualized in Fig. 1. Each section begins with some general description of numbers of participants who had discontinued or recommenced PrEP, or engaged in CLAI, before continuing with more detailed analysis with quotations. While we acknowledge that the qualitative nature of these data and are not intending to present this information as representative, providing description of these numbers adds much needed contextual detail to participants’ overall patterns of PrEP use.

### Discontinuing PrEP

Just over half of participants (n = 22) had discontinued PrEP at least once. Periods of not using PrEP after discontinuing it generally lasted between 3 and 6 months, with a few up to 3 years. Periods of more than one year off PrEP were generally reported by those who entered monogamous relationships, as well as two participants who experienced side effects.

#### Reasons for Discontinuing PrEP

Participants reported various reasons for discontinuing PrEP and their reasons often overlapped. Most commonly, participants indicated engaging in less or no sex, or not having sex that put them at risk of HIV acquisition:

I stopped [PrEP] for about six months for no other reason than I just lost all interest in sex. I always tend to go quiet in wintertime. So last winter I stopped taking it because there’s no point (35 years, White European).

Similarly, another participant commented:

I stopped when the [COVID-19] lockdown became serious and my rationale was financial. I was thinking: “there’s no chance I’m going to have sex for at least three months. Why spend $40 a month having it?” (60 years, White European).

For both participants quoted above, discontinuing PrEP coincided with periods of reduced sex. For the former participant, reduced sexual activity was characterized as a regular, seasonal event. In contrast, the latter participant described restriction measures associated with COVID-19 as limiting opportunities for sex, making PrEP not worth the financial outlay. Importantly, both participants characterized their discontinuation as temporary and left open the option of recommencing PrEP.

Periods of more than one year off PrEP were generally reported by those who entered monogamous relationships: *We just stopped using PrEP full-time. Gave it up and decided to become monogamous. And we’ve continued in that vein* *(40, White European).* Although this participant did not necessarily rule out recommencing PrEP should his relationship change, PrEP was considered unnecessary while his relationship remained monogamous. Finally, others described concerns over toxicity and adverse side effects as underpinning their decision to discontinue PrEP.

After six months, they were doing kidney-function tests and my readings were going down so [the healthcare worker] said “we need to monitor that more closely.” Eventually they said “Alright, this is not for you” (50, White European).

#### Methods of Discontinuing Pills

Most participants simply stopped taking their pills when they wanted to discontinue PrEP and retained their leftover pills: *I just stopped and kept whatever I had left (63, White/European).* Retaining pills suggested they either expected they might return to PrEP at some point or saw no benefit in continuing medication. However, a few continued taking their pills until they ran out. Generally, this coincided with a realization that they had been sexually inactive and saw no future sexual prospects at the time of discontinuing. A few participants who were not having sex said that they gradually took fewer pills per week until eventually discontinuing altogether: *But it’s like, maybe gradually decreasing until the point that I just gave up because I wasn’t really having sex* (34, Northeast Asia).

### CLAI After Discontinuing or Suspending PrEP

Of the 34 who had discontinued or suspended PrEP at least once, most (n = 22) did not have sex or did not have CLAI while not on PrEP. However, 12 participants had CLAI with casual or fuckbuddy partners while not on PrEP (after discontinuing or during suspension). Of these 12, eight had CLAI after discontinuing, two had CLAI during involuntarily suspension, and two during voluntary suspension. Of the eight who had CLAI after discontinuing, six had CLAI on multiple but dispersed occasions and two had CLAI only once, which instigated recommencing PrEP.

Both of the two who had CLAI when they voluntarily suspended did not take PrEP with them while travelling to see family but had unanticipated sex with casual partners. One had CLAI only once and one had CLAI on a few occasions within a week. Of the two who had CLAI when they involuntarily suspended, one lost his luggage with PrEP in it at an airport while travelling and then had CLAI once, and one was experiencing significant mental health concerns and was unable to take PrEP during this period but continued to have sex on a few occasions over a few weeks. The sub-sections below provide more detailed description of participants’ reasons for not having recommenced PrEP and not using condoms during periods off PrEP.

#### Reasons for Not Having Recommenced PrEP Prior to CLAI

Of the participants who had CLAI only once or on a few occasions within a brief period, their main reason reported for not having recommenced PrEP prior to engaging in CLAI was that the sex events were unanticipated. These CLAI events acted as an indicator to these participants that they should recommence PrEP.

I think I did have a bit of sex after I stopped. It just sort of happened, I wasn’t expecting it. I was on a trip to Melbourne and I had sex during that trip. And then it was like, “Okay, if I wanna have anal sex, I need to make sure that I’m on PrEP”. (54 years, White European)

The participants who had CLAI on multiple but dispersed occasions after discontinuing reasoned that not only were the events unanticipated, but they had not already recommenced PrEP because they did not view themselves as engaging in frequent enough risk to warrant recommencing. This is because the sex events were infrequent and spread across time.

I didn’t think I needed it [PrEP] ‘cause I wasn’t having sex for most of last year. It was like very few sporadic times so I just thought, “Oh, no, if it happens, if I’m sporadically having sex, I’ll use a condom because I just don’t want to have to take a pill every day.” 30 pills for one day that I might benefit from using it. (28 years, White European)

This participant had CLAI on three occasions over a period of approximately nine months. He had discontinued PrEP due to a relationship ending and reported that he had lost interest in sex. Although he believed he would use condoms if he did have sex, this did not eventuate.

#### Reasons for Not Using Condoms After Discontinuing or Suspending PrEP

Some of the 12 who had CLAI reported that after discontinuing or suspending PrEP they generally would have considered using condoms when they had sex, but nonetheless had occasions of CLAI. In these instances, they reported that they got caught in ‘the heat of the moment’: *They were casual encounters with people I hadn’t met before. These were chance encounters, and I was enjoying the moment. I was caught up in the heat of the moment (54 years, White European).* A few participants also reported acquiescing to pressure from partners requesting CLAI.

When you’re on PrEP, you’re feeling more liberated, and you’re like, “You know what? Let’s do it without it [condoms]!” But, once PrEP was gone, I wasn’t so cavalier. I was like, “Yeah, okay, we’re bringing back condoms now.” But I feel like I am not very good at putting my foot down, and I feel like sometimes guys pressure you into not using them, or say they’re on PrEP so it doesn’t matter. And sometimes like you don’t wanna be like the rude person. (28 years, Northeast Asia)

This participant’s description indicates he experienced some difficulty negotiating condom use in a context of PrEP becoming a more normative HIV prevention choice among GBM in Australia (see [28]). His description suggests that since the proliferation of PrEP, new expectations and norms surrounding condom use have emerged, with those preferring condoms facing pressure to acquiesce to sex without condoms lest they face rejection.

However, keeping in mind that condom use dramatically decreased for most participants after PrEP initiation, for the most part condoms were generally not viewed favorably in lieu of PrEP. Participants instead reported adopting a range of strategies when not using PrEP or condoms. First, one theme identifiable in participants’ accounts of CLAI after discontinuing was familiarity with sex partners. As the following participant reported, limiting sex to someone he ‘knew well’ ensured HIV prevention coverage via their familiarity with a partner’s use of PrEP if HIV-negative or undetectable viral load if living with HIV.

My sex life was very unpredictable. If I did have sex, it would always be with someone I knew well. In my mind, I was like, “Well, the safest thing to do would be ideally go back on PrEP myself but, if I can’t do that, I can pick guys I talk to a lot that I know are on PrEP or are undetectable, and basically just knock everyone else out of consideration.” I also didn’t think I needed to be on it all the time when I wasn’t having such a regular sexual habit. (34 years, Northeast Asia)

Second, other participants adopted a range of risk reduction strategies during CLAI events, such as withdrawing before ejaculating, taking the insertive position, or asking casual partners about status. However, these strategies were often applied inconsistently. For example, one participant described three occasions of CLAI over a period of approximately nine months, mostly after nights drinking at bars.

The first occasion I didn’t think was as bad because I was the top and so I was like, “Oh, I s’pose there’s lower risk.” The second I was a bottom, and so I knew I was at greater risk… At that time, he told me he was on PrEP so we didn’t need to use a condom. I think I tried to rationalize it being like, “Oh, well, if he’s on PrEP, it’s very low-risk… The third one we were about to have sex but he was like, “Oh, I can’t keep it up if I wear a condom.” So I just said, “Oh right, whatever”. (28 years, White European)

Though he described some forms of HIV risk reduction in these encounters, this was applied inconsistently and without good familiarity with the partners involved. Also, although he ultimately agreed not to use condoms upon the requests of partners, elsewhere in his interview he made it clear he disliked condoms anyway, which may explain why he easily acquiesced.

Finally, some participants reported that rather than adopting any risk reduction strategies, they made assumptions about a casual partner’s HIV status or use of PrEP without prior knowledge or discussion, and as the following excerpt suggests, drew on probabilities of other men’s PrEP use in their social/sexual circles.

I did go for a weekend in [city] to see friends and did have sex without being on PrEP but then the assumption was everybody else was. So in my eyes it wasn’t that big a risk. (55 years, White European)

### PrEP Recommencement

Of the 34 who had discontinued or suspended PrEP at least once, the majority recommenced using PrEP daily. Participants mostly recommenced PrEP because they wanted to have more sex or anticipated having sex more frequently in the future.

I was bored and horny but also the restrictions were lifting and more people were understanding where COVID hot spots were and perhaps thinking that people were safer. So I went back on with the full intention of hooking up (32, White/European)

Other reasons included: after engaging in CLAI, taken as an indication that they should recommence; ending a relationship and reengaging with sex with casual partners; and changing a relationship status from monogamy to nonmonogamy.

Of the 12 who had CLAI after discontinuing or suspending PrEP, all subsequently recommenced PrEP; 10 recommenced daily and two recommenced with event-driven dosing. The majority of the 12 had not considered taking PrEP event-driven when they had CLAI because they: had never heard of it or had a very vague understanding that a non-daily dosing strategy existed; had heard of it but had not been exposed to enough information to feel confident pursuing it; or were accustomed to or preferred daily PrEP and saw little need to pursue it.

#### Methods for Recommencing Pills

Many participants, including the 12 who had CLAI after discontinuing or suspending PrEP, did not know that taking a loading dose of two pills two to 24 h prior to sex to recommence daily PrEP provides adequate protection, as was recommended in Australia’s PrEP guidelines at the time of data collection for this study [28]. Several participants believed that when re-commencing, they needed to accumulate PrEP in their bodies for at least several days before they would be protected by it. This understanding reflected what they recalled learning when they first initiated PrEP, which for most was anywhere between seven days and one month. However, this recollection was often vague.

*I was aware that the more you take it the better when you’re restarting, but that’s about it. Like the more days you’ve been taking it daily the better. And I believe it’s about a week you’re supposed to take it to get to sort of that maximum level of safety (34 years, White European)*

This reflected previous PrEP prescribing guidelines in Australia that recommended GBM take PrEP for several weeks before they were considered protected [29]. By the time of data collection for this study, public promotion of the two-pill loading dose for recommencing PrEP had only recently begun and it is unsurprising that their beliefs reflected previous guidelines.

Many participants recalled that they either chose an arbitrary day to recommence PrEP in anticipation of having sex in the near future, or that recent re-engagement with sex indicated to them that they should recommence. To recommence PrEP, participants usually continued using the pills they had leftover prior to making an appointment with a doctor to ‘formally’ recommence. This was because many had not had sex when they took time off PrEP and saw no need to see a doctor until they needed new pills.

## Discussion

There was considerable diversity in participants’ patterns of PrEP use over time. Specifically, discontinuing or suspending PrEP formed only one component of participants’ broader ongoing use and their pathways of using PrEP were unique and sometimes intricate. Also, the data we captured presents only a snapshot of participants’ PrEP use up until the time of their interviews—it is likely at least some of the participants would have further changed their use since that time. This study is one of a few that has mapped how GBM’s PrEP use changes beyond reporting on one-off occasions of discontinuation (see, for example, [3, 9, 18]). Several participants had discontinued or suspended PrEP on multiple occasions, usually for periods of between three and six months. Even if they had no future intention of returning PrEP at the time of discontinuing, most participants nonetheless did recommence PrEP. Participants usually made accurate decisions about discontinuing or suspending PrEP based on expectations about sexual behavior in the future. However, 12 participants did engage in CLAI during discontinuing or suspending PrEP.

Researchers have argued more data are needed to understand how GBM’s sexual behaviors change after discontinuing PrEP, including how they make decisions about other HIV prevention strategies [16]. The concept of ‘prevention-effective adherence’ includes not just whether PrEP is adhered to during sexual events, but also whether other risk reduction strategies are adopted [20]. Our study provides valuable insight into why and how CLAI occurs after GBM discontinue or suspend PrEP. Participants had not recommenced PrEP before engaging in CLAI because the sex events were unanticipated, and despite some engaging in CLAI on several occasions, there was a perception that they had not engaged in frequent enough risk to warrant recommencing. At the same time, condoms were not a preferred choice during sex events. Indeed, some participants reported that even prior to commencing PrEP they already preferred CLAI or had been increasingly engaging in CLAI, as has been found elsewhere [31]. Nonetheless, such condom attitudes likely reflect now well-known shifts in HIV prevention norms—PrEP has allowed GBM to have CLAI without fears of HIV transmission [32–36], and PrEP uptake has increased concurrently with declines in condom use [28]. This may explain why returning to condoms after discontinuing or suspending PrEP was a difficult prospect for participants. Condoms may not be perceived as a viable option for many GBM who discontinue or suspend PrEP, but nor may returning to daily PrEP if they engage in infrequent and episodic CLAI over longer periods of time.

Our study also provides valuable insight into the strategies that are adopted in lieu of PrEP and condoms after GBM discontinue or suspend PrEP. During sex events, some participants did adopt some risk reduction strategies, but these strategies were applied inconsistently. Also, some participants either assumed or trusted that their sexual partners were on PrEP or had undetectable viral load. There is an important distinction between ‘assumption’ and ‘trust’ when assessing HIV risk. A few participants had CLAI with regular fuckbuddy partners who were familiar and trusted. Through the development of familiarity and trust, some GBM can have accurate knowledge of a regular sexual partner’s HIV status [36, 37], which has been shown to make CLAI safer [39]. Our data show that employing familiarity and trust of HIV status to reduce HIV risk likely also applies to knowing a partner’s PrEP use and viral load. In these instances, effective HIV prevention is more likely to occur even in the absence of an individual’s own use of PrEP [20], but ongoing and honest communication are important for this knowledge to remain accurate and trustworthy [40, 41]. In some circumstances, such as when sexual partners are unaware of their HIV status or level of protection provided by PrEP, such strategies may be less viable. During CLAI, some participants assumed their partners were HIV-negative, on PrEP, or had undetectable viral load without prior discussion, and these CLAI events are likely to be riskier for HIV transmission. So, in the absence of an individual’s own use of PrEP and if condoms are not preferred, accurate and ongoing knowledge of a partner’s HIV, PrEP, or undetectable viral load status can be one tool for reducing HIV risk, but it is likely to be most effective only with familiar or trusted regular partners. It is less clear how effective these methods (i.e. relying on another’s use of PrEP or undetectable viral load) may be for casual sex contexts, particularly given the lack of clarity regarding accurate assessments of sexual partners.

GBM who discontinue or suspend PrEP but remain sexually active may require guidance about non-condom-based HIV prevention during periods off PrEP. Updated health promotion initiatives might consider guiding GBM in identifying the circumstances in which reasoned trust may be utilized for employing HIV risk reduction. Promotion of event-driven PrEP may be another HIV prevention tool. Event-driven PrEP involves taking pills around the time of a sex event [42] and has been found to be more attractive to those who have infrequent HIV risk [23]. However, to effectively use event-driven PrEP, individuals need to ensure they have access to pills at least two hours prior to sex, which some participants found difficult when sex was entirely unanticipated. Also, the majority of participants only had familiarity with daily PrEP and had never seen or sought out information about event-driven PrEP. Though event-driven PrEP received endorsement in clinical guidance in Australia in September 2019 [29], large-scale promotion of it in the community only occurred in early 2021 [43], so it is perhaps unsurprising many participants had never considered it. As such, continued efforts to scale up promotion of event-driven PrEP may benefit some GBM who discontinue or suspend PrEP and subsequently engage in occasional CLAI. Such efforts should be broadly targeted to all GBM, including those who prefer daily PrEP, to raise awareness of HIV prevention options should their circumstances change.

Our results on the reasons participants provided for discontinuing PrEP support previous research, including changed perceptions of risk [5, 12, 17], experiences of side effects [12, 14], concerns about toxicity [14], and cost [5, 12]. However, in our study, cost was a relative rather than absolute issue, mostly weighed against participants’ infrequency of sex. Had they been having more sex, few participants reported that they would have found the publicly subsidized price too high. However, our sample was largely GBM of higher socio-economic status (evidenced through a combination of their education and employment status, though notably we did not collect income information). Recent research has found that PrEP in Australia may be too costly for those who do not have access to Medicare, so there certainly may be some GBM who may find PrEP in Australia too expensive [44]. Nonetheless, no participants in our study reported that they discontinued PrEP because they were no longer able to access it. This contrasts other research that describes financial and other systemic barriers, such as insurance coverage (in the USA) and lack of appointment availability, as reasons GBM discontinue PrEP [5, 12, 14, 16]. Our results indicate that when public health systems have features that make PrEP accessible (such as publicly subsidized pills, networks of sexual health clinics that provide free testing, and allowances for all doctors to prescribe), GBM are more likely to remain on PrEP for as long as they believe they are at risk of HIV acquisition [45].

Acknowledging the range and diversity of GBM’s PrEP use over time centralizes *ongoing* use, which has implications for implementing clinical care and health promotion. PrEP guidelines in Australia and internationally state that when an individual wishes to recommence PrEP, they should first have repeat HIV testing in case they have acquired HIV during the time they were not taking PrEP [46]. Guidelines typically do not differentiate between people who have had sex that might put them at risk of HIV acquisition and those that have not. In our sample, many participants saw no need to see a doctor for HIV testing before recommencing PrEP because they had not had sex during periods off PrEP. Furthermore, some had frequently discontinued and recommenced, sometimes within a short space of time. The current HIV testing requirement for PrEP recommencement may act as a barrier to efficiently recommencing PrEP if it seems unnecessary or too burdensome to some GBM. Of course, due to the regular updating of PrEP information, the recommendation for HIV testing should remain for those who have had long periods off PrEP. Other ways clinical care and health promotion might more appropriately support GBM to safely and efficiently change their PrEP use include: encouraging GBM to preserve some pills after discontinuation should risk recommence (while being cognizant of pill expiry); supporting GBM in recognizing changing risk and recommencing PrEP when appropriate and prior to engaging in HIV risk; promoting event-driven PrEP for GBM who discontinue PrEP but might have occasional CLAI after discontinuing; and raising awareness of how to recommence PrEP safely using the two-pill loading dose, if recommencement becomes relevant. The latter strategy may be of particular benefit because few participants in our study knew about the two-pill loading dose for commencing daily PrEP and instead assumed recommencing PrEP required several weeks of pill-taking before being effective, which may be a barrier to recommencement.

### Limitations

We mainly recruited highly educated men of White European cultural/ethnic background, who had a gay sexual identity, who were well connected to sexual health services prior to initiating PrEP, and who had excellent health literacy. Future research should incorporate experiences from a sample of GBM more diverse in cultural/ethnic background, sexual identity, and levels of engagement with sexual health services. Due to the recruitment strategy, the majority of participants had previously been involved in a clinical trial of daily PrEP and were thus likely part of the early and most motivated wave of PrEP adopters. Also, we did not capture experiences of GBM who had never discontinued PrEP nor those who initiated PrEP with a non-daily dosing regimen. There was no formal member checking implemented during analysis of this project, presenting a limitation to data validation. However, to provide participants input and validation opportunities, the interviewer continuously checked with participants that interpretations of participants’ own descriptions were appropriate throughout interviews. Australia is a setting with lower-cost access to publicly-subsidized PrEP. The results from our study may not be generalizable to other contexts, particularly in countries in which PrEP is not readily available, lower-income countries with limited resources, and countries with higher levels of HIV stigma and homophobia.

## Conclusion

In this qualitative study of GBM who had changed their PrEP use since initiating, we found substantial variation in patterns of PrEP use, and discontinuation formed only one part of broader ongoing PrEP use. Participants’ reasons for changing their PrEP use mostly centered on perceived changes to risk. Among those who discontinued, experiences of side-effects were mentioned, as well as concerns for toxicity and cost, but these were concerns relative to participants’ infrequency of sex. We have provided insight into the contexts in which CLAI occurs when GBM discontinue or suspend PrEP. Most participants displayed self-efficacy in managing PrEP use in accordance with their level of HIV risk. However, after discontinuing or suspending PrEP, several participants reported CLAI with both casual and fuckbuddy partners, mostly in the context of sex events that were unanticipated. While some participants adopted other risk reduction strategies during these CLAI events, others made assumptions about partners’ HIV, viral load, or PrEP status, potentially representing contexts of HIV risk.
